# Supplementary figures and images for: Spot-fire distance increases disproportionately for wildfires compared to prescribed fires as grasslands transition to Juniperus woodlands
Source: PLoS One. 2023 Apr 11;18(4):e0283816. doi: 10.1371/journal.pone.0283816 (PMC10089345; doi:10.1371/journal.pone.0283816)

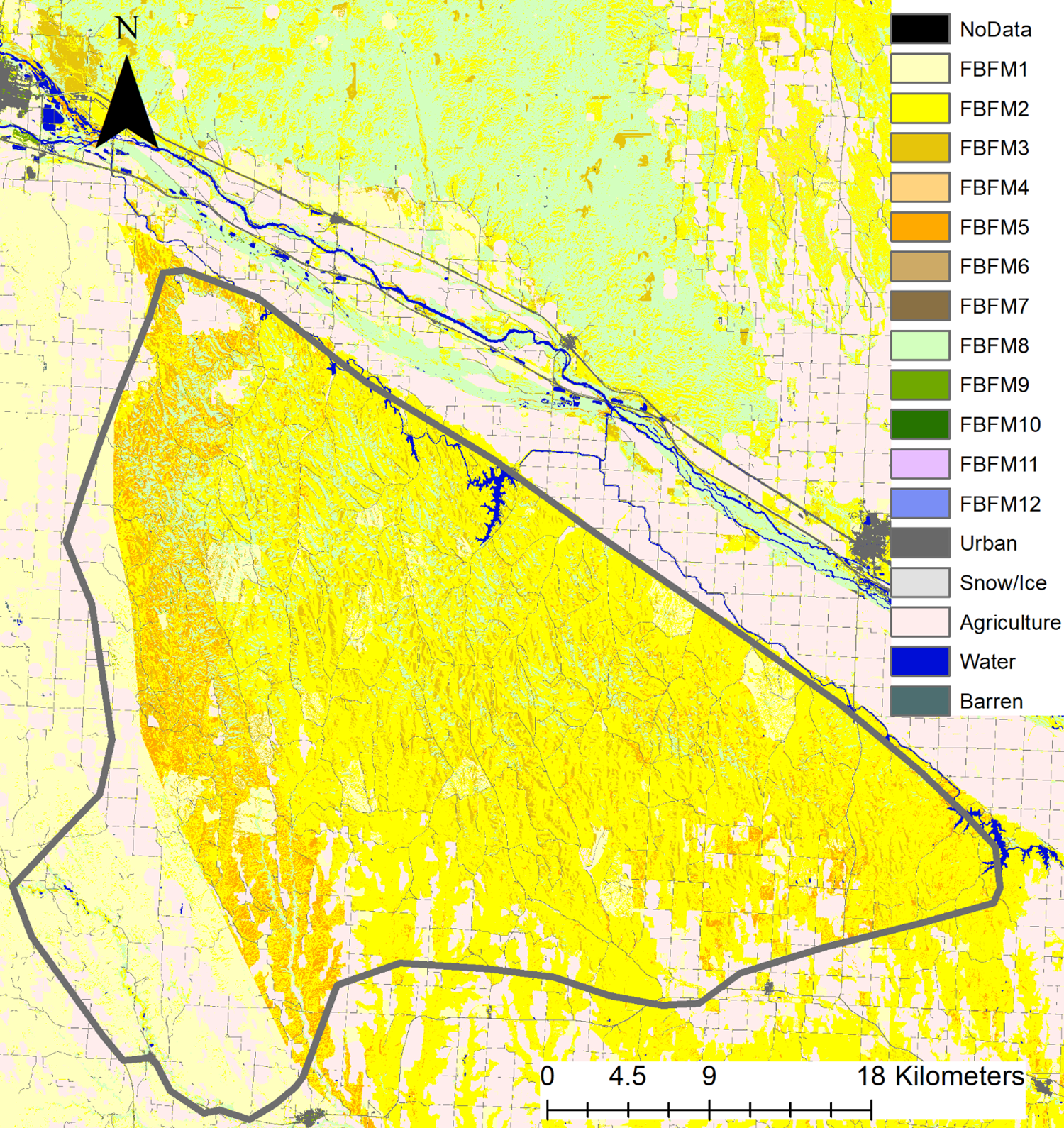

Supplement: S1 Fig — (TIF) [file pone.0283816.s001.tif]
